# Navigating evidence-informed decision making for arboviral disease control in Pacific Island countries

**DOI:** 10.1186/s44263-026-00289-4

**Published:** 2026-05-26

**Authors:** Anaseini Ratu, Katherine L Anders, Karin Leder

**Affiliations:** Infectious Diseases Epidemiology Unit, Planetary Health Division, School of Public Health and Preventive Medicine, Monash University, Melbourne, Australia

**Keywords:** Evidence-informed decision-making, Pacific Island Countries, Health policy, Arboviral diseases, Climate-sensitive diseases, Knowledge translation, Public health, Impact evaluation

## Abstract

Arboviral disease control is a priority for Pacific Island countries (PICs), where dengue outbreaks occur frequently. This perspective applies the World Health Organization (WHO)’s evidence-informed decision making (EIDM) framework to examine opportunities and challenges in generating, translating and using evidence to guide arboviral disease control in PICs, using the Wolbachia mosquito release intervention as a case study. The implementation of Wolbachia in several Pacific countries was achieved in the absence of a comprehensive evidence ecosystem, highlighting gaps in EIDM processes. In particular, limited local evidence on intervention effectiveness, combined with challenges in disease surveillance, data accessibility and entomological monitoring, has hindered robust evaluation of public health impact and decisions on scale-up and sustainability. Opportunities exist to strengthen EIDM through pragmatic use of existing surveillance data, time-series analysis and serological studies to assess impact. An untapped reservoir of tacit evidence can be harnessed through culturally appropriate methodologies, such as *Talanoa*, to contextualise scientific data and address gaps in qualitative research. Closer connections between evidence generators and users can facilitate more effective collective decision-making in Pacific settings. The WHO EIDM framework is useful for Pacific populations to chart pathways towards improved arboviral disease control, with pragmatic considerations of its application in Pacific contexts. Enhanced collaborations and transdisciplinary programs offer further opportunities to align interventions with regional health priorities for integrated EIDM. Policy dialogues using culturally appropriate approaches and leveraging the advantage of simpler systems can greatly improve the efficiency of knowledge translation pathways for EIDM in PICs.

## Background

The emergence and re-emergence of dengue and other arboviral diseases with epidemic potential has impacted communities and health systems in Pacific Island countries (PICs) significantly [1]. Countries have recognised the urgent need to strengthen prevention and control efforts to reduce the health, economic and societal burden of outbreaks in this region [1–4]. PICs include a diverse group of 22 island nations and cultures across the Pacific Ocean with populations that range from 50 people to 10 million people [5]. The frequency and intensity of dengue outbreaks has increased regionally with concurrent emergence of other mosquito-borne viral diseases including chikungunya and Zika [1, 6–8]. In 2025, dengue outbreaks were declared by health ministries in nine PICs, with 40,000 cases of dengue-like illness reported to the Pacific Syndromic Surveillance System, and more than 20,000 laboratory-confirmed dengue cases, 4000 hospitalisations and 21 deaths, mainly in children [9]. *Aedes aegypti* mosquitoes are the primary vectors of dengue transmission and are prevalent throughout PICs [10, 11].

Strengthening the implementation and effectiveness of interventions to prevent and control arboviral disease outbreaks is a global, regional, country and community priority [4, 6, 12, 13]. The World Health Organization (WHO)’s Western Pacific regional action plan for dengue prevention and control (2016) emphasises evidence for action with evaluation of new interventions soon after their adoption [1]. Innovative approaches for dengue control have been implemented in the Pacific such as biological methods including Wolbachia mosquito releases and the sterile insect technique (SIT) [11]. Introduction of the Wolbachia intervention in Pacific countries is indicative of country commitments to embrace novel approaches and expand existing efforts to contain projected rises in disease occurrence [6]. Pacific health leaders have emphasised the importance of data quality and availability for decision making, and the need for investment in scaling up public health interventions which requires prior evaluation [12]. Evaluations of public health interventions are typically conducted by or in conjunction with the program implementers and are commonly process evaluations reporting operational indicators and program outputs. While process evaluations can provide valuable insights into local contextual factors influencing intervention outcomes that are important for informing future implementation, impact evaluations reporting measurable changes in health outcomes are also critical for decision-making. The assessment of health outcomes relies on either analyses of existing clinical and public health surveillance data or primary data collection within a research framework, which often relies on external funding support and may be hindered by externally-driven research agendas and constrained research governance capacity within PICs [14]. Indeed, a recent scoping review of health research in four PICs between 2014 and 2024 found that only 10% of studies reported evaluations of the effectiveness or impact of interventions or programs [12, 15].

Pathways for generating the most relevant evidence and facilitating its use in decision making are not well defined and there is a recognised gap between evidence generation and translation [14]. This impacts the adoption and expansion of innovative interventions such as Wolbachia-based dengue control, which has been implemented in four PICs. Evidence of the successful introduction of Wolbachia bacteria into local mosquito populations to prevent dengue virus transmission in Pacific intervention sites has been demonstrated from results of long-term entomological monitoring [10]. Demonstration of the epidemiological effectiveness of the Wolbachia intervention in reducing the incidence of dengue and other arboviral diseases in Pacific implementation sites is yet to emerge as there has not been an evaluation of disease outcomes [10]. This absence of evidence for the public health impact of the Wolbachia intervention in Pacific sites is a limiting factor in decision-making on scaling this intervention and planning for long-term disease control strategies [10, 12]. Furthermore, the ability to inform Pacific populations involved in and affected by the Wolbachia intervention, on its efficacy, is currently limited, and is important for accountability and strengthening trust in health programs.

Existing regional and national guidance for arboviral disease control is informed by global guidance on mosquito control, [16] surveillance and clinical management [4] without emphasis on how evidence for interventions might be generated or utilised. Few frameworks exist globally that outline how health data and research evidence can be translated to inform policies, programs and decisions regarding the uptake of new interventions [17] however WHO’s evidence informed decision-making (EIDM) model offers guidance on how this might be achieved [18]. This WHO EIDM framework outlines types of scientific evidence and describes how these might be integrated with contextual considerations to enhance effectiveness, efficiency, and equity in decision making for health [18]. We use the WHO EIDM framework to assess opportunities and challenges for increasing the use of evidence in arboviral disease control, utilising insights from introduction of the Wolbachia intervention in Pacific countries.

## The WHO EIDM framework and its components

WHO proposes EIDM as a ‘systematic and transparent approach that applies structured and replicable methods to identify, appraise and apply evidence across decision making processes’ [18]. The components of EIDM constitute an ‘evidence ecosystem’ that includes both the process of generating and synthesising different types of evidence, as well as the application of evidence [18]. This is facilitated by knowledge translation pathways that support the use of research evidence in developing policies or directing actions for improved health outcomes [18].

Overlapping and complementary categories of evidence are distinguished: scientific (from formal research and data analysis) versus tacit evidence (from informal expertise and community input); and globally-generated versus local, context-specific evidence [18]. The type of evidence required is dependent on the specific question or issue being addressed. Evidence generation occurs in three stages - evidence inquiry, synthesis, and product creation - while the application of evidence occurs throughout the policy-action cycle: understanding the problem, designing solutions, and achieving impact [18].

### Overview of the Wolbachia intervention in PICs

The implementation between 2018 and 2023 of Wolbachia-based dengue control by the Ministries of Health of four PICs, in partnership with the Australian-based *World Mosquito Program* (WMP), was a novel approach for vector-borne disease control in the region [10]. This innovative disease control strategy inhibits the capacity of mosquitoes to transmit dengue and other arboviral diseases by introducing the symbiotic insect bacterium Wolbachia into the local *Ae. aegypti* mosquito population [10]. Presence of the bacteria in *Ae. aegypti* inhibits the transmission of arboviruses thereby reducing the occurrence of disease in humans [19]. Wolbachia releases in the Pacific commenced in Fiji, Vanuatu and Kiribati in 2018, focused on high density urban areas, and were conducted with donor support [10]. These occurred through area-wide deployments in pre-determined release sites, requiring community support for weekly releases over a period of 2 to 5 months [10]. Long term monitoring of the intervention through entomological assessment of Wolbachia frequency in local *Ae. aegypti* populations has shown successful establishment of Wolbachia in major urban areas of Fiji, Kiribati and Vanuatu [10]. New Caledonia has also successfully implemented Wolbachia with high community support [20]. Public health impact in PICs is anticipated based on results from other global sites but is yet to be formally evaluated in these settings [10].

### Evidence generation for Wolbachia-based dengue control

#### Scientific evidence

Table 1 summarises existing sources of research evidence for the Wolbachia intervention in PICs, with reference to the three phases of evidence creation in the WHO EIDM framework and categorised by global versus local evidence.

**Table 1:** Evidence available to inform decision-making for implementation of Wolbachia-based arboviral disease control in PICs

Evidence	Global evidence	Local (PICs) evidence
**Evidence Products** Tertiary research	No global products. Country-specific public health guidelines in Brazil [21] and Indonesia [22].	None
**Evidence Synthesis** Secondary research	Several systematic [23, 24] and qualitative reviews [25] and modelling studies [26].	None
**Evidence Inquiry** Primary research	Substantial. One RCT in Indonesia, [27] quasi-experimental, [28] observational [29–33] and economic modelling [34] studies from Asia-Pacific and South American sites. Implementation studies reporting entomological outcomes [10, 35, 36].	Limited. Implementation studies in Fiji, [10, 36] Vanuatu, [10] Kiribati [10] and New Caledonia [35]. Observational studies [8, 37] and a review [3] describing arboviral disease outbreaks in Pacific countries. *Surveillance data. *Routine program data including community surveys.

*Not publicly accessible

**Fig. 1:**
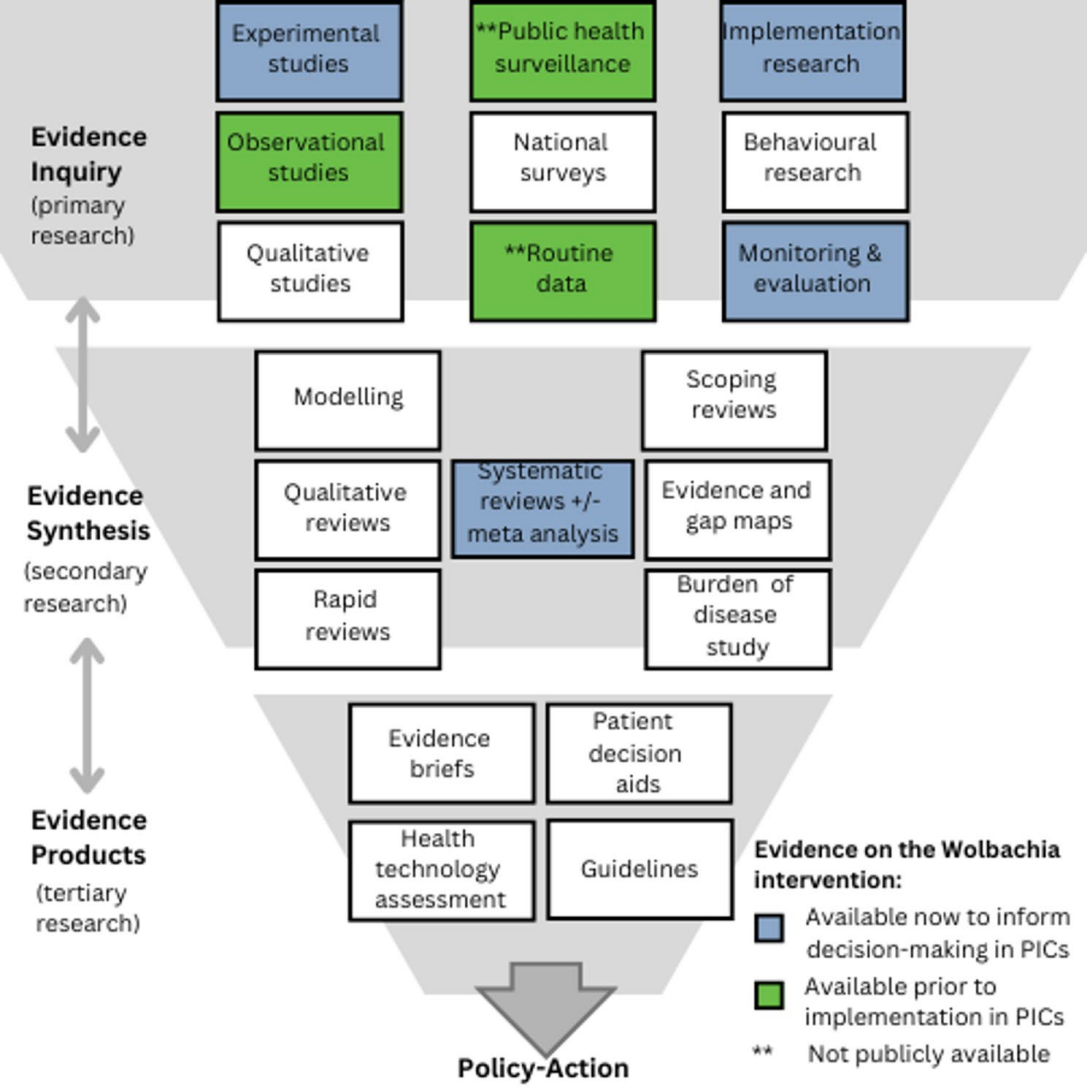
Evidence generation funnel for the Wolbachia intervention in PICs, adapted from the WHO EIDM guide 18

Figure 1. shows the types of available evidence relevant to Wolbachia implementation in the Pacific region and their availability relative to when Wolbachia was introduced.

**Fig. 2:**
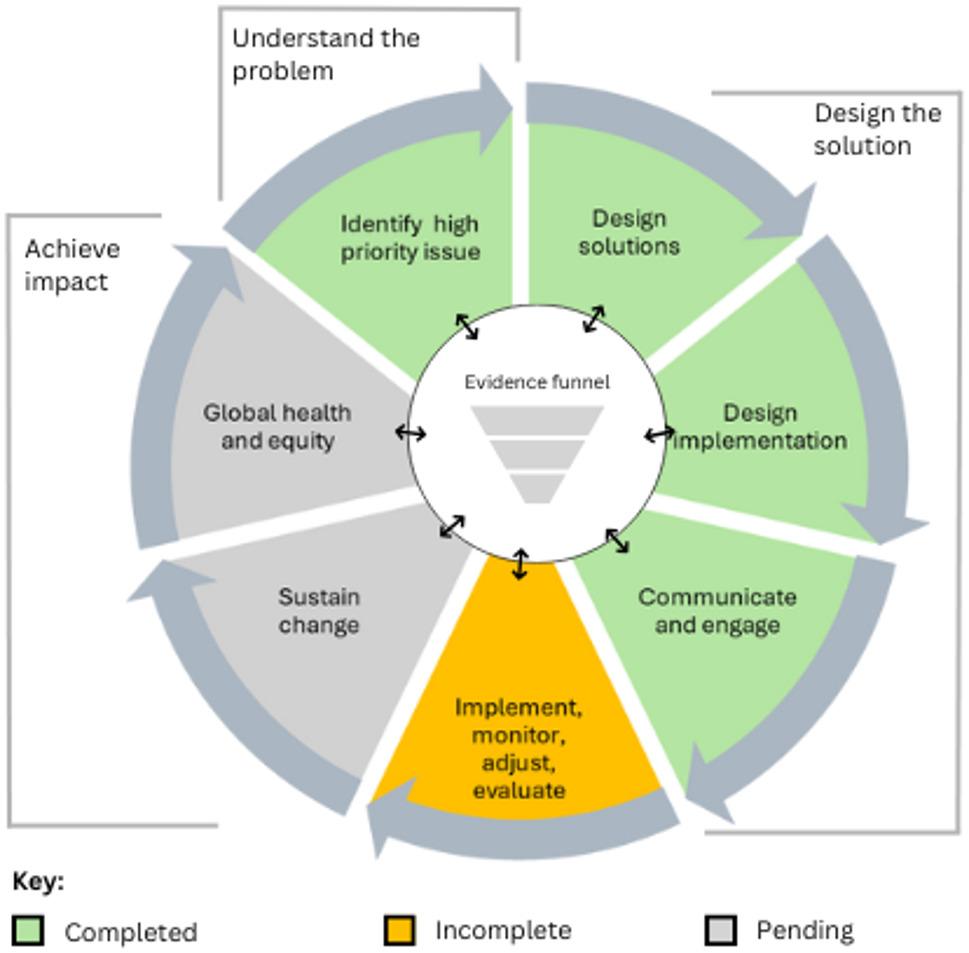
Evidence ecosystem for impact for the implementation of Wolbachia-based dengue control in PICs, adapted from the WHO EIDM guide [18]

Figure 1. shows that synthesised evidence was not available pre-implementation and identifies existing gaps in primary research. This includes qualitative and behavioural evidence that could contextualise quantitative measures of impact, inform future adaptation and sustain change. In the WHO EIDM framework, this evidence funnel (Fig. 1) is used throughout the policy/action cycle to inform each step, from understanding the problem to designing solutions and achieving impact, as shown in Fig. 2 [18]. Fig. 2. illustrates this cycle for the Wolbachia intervention in PICs as informed by the evidence funnel in Fig. 1. Each step of the cycle is informed by the evidence funnel (Fig. 1) towards EIDM for impact 18.

Figure 2. emphasises the current incompleteness of the policy/action cycle to sustain change and achieve optimal impact through Wolbachia-based dengue control in Pacific countries. The identification of dengue and other arboviral diseases as a high priority public health issue, was informed by primary data from public health surveillance and routine entomological monitoring that demonstrated a high baseline dengue disease burden and the presence of *Ae. aegypti* as the primary vector [10]. Solution design was based on evidence from successful Wolbachia implementation in other countries, and an understanding of *Ae. aegypti* local ecology and dengue epidemiology from observational studies and routine program data [10]. Implementation and monitoring were also informed by data from other global release sites, local monitoring data and behaviour research from community and stakeholder engagement [10]. This design stage was led by WMP staff in consultation with ministry of health partners and local project staff [10] but lacked integration into the broader policy/program cycle. Stakeholder and community engagement was a key component of the Wolbachia implementation programs, and evidence generated from community surveys showed high acceptance of the Wolbachia intervention following engagement [10]. More contextual understanding of a range of perspectives would be valuable. Long-term entomological monitoring of Wolbachia ceased in Fiji, Vanuatu and Kiribati when project cycles for funding support ended and disease monitoring to generate measures of public health impact are yet to emerge [10]. Hence, adjustments such as scale-up across islands in each Pacific site have not been conducted. Furthermore, the role of Wolbachia based dengue control in the Pacific as part of integrated vector control strategies remains unclear.

Substantial global scientific evidence for the Wolbachia intervention, including a systematic review [23] and modelling study, [26] emerged post implementation in PICs [25] (Fig. 1), however it is clear that more local PIC evidence is still needed (Table 1). Global primary research includes one cluster randomized trial from Indonesia [27, 30] and quasi-experimental studies in Brazil, [29, 33] Colombia [32] and Malaysia [33] that have shown reduced dengue incidence following Wolbachia establishment in local mosquito populations. In Pacific Wolbachia sites, published results show the successful introgression of Wolbachia into local mosquito populations in five Pacific cities at between 19 and 33 months after the end of releases [10]. However, an understanding of the long-term stability of Wolbachia in the Pacific sites has been limited by the cessation of the entomological monitoring of Wolbachia in local *Ae. aegypti* populations that was externally supported for Wolbachia releases, and robust local efficacy data describing impacts on public health outcomes have not yet been compiled or published [10]. This limits the ability to maximise the impact of this intervention through the application of evidence to sustain change and promote health equity (Fig. 2) [10, 18]. Existing data sources such as public health surveillance data might be used for evaluations to ‘achieve impact’ in the evidence ecosystem (Fig. 2.) but have not yet been applied for this purpose. An evaluation of the cost-effectiveness of the Wolbachia intervention in Fiji and Vanuatu was undertaken as part of a donor-funded program evaluation, but is also unpublished [38]. The following section discusses evidence gaps that currently exist and opportunities to address these in the Pacific context.

### Sources of scientific evidence

#### Program data for monitoring and evaluation

Program data on monitoring and evaluation of health interventions varies in scope and availability for EIDM by decision makers [39, 40]. Limited program data is publicly available for the Wolbachia intervention in PIC intervention sites and impact evaluations rely on the availability of routine surveillance data [10]. Implementation data has been shared with local ministry of health stakeholders however long-term monitoring was affected by the COVID-19 pandemic which coincided with the end of donor support for program activities in PICs [10]. Strengthened local ownership of program data and the dissemination of program outcomes among stakeholders and the public may improve local perceptions, and promote participation by these groups in knowledge translation pathways and decision-making around any future scale-up.

#### Public health surveillance for arboviral diseases

The public health surveillance of arboviral diseases is well established in PICs with strong regional networks for surveillance through the Pacific Public Health Surveillance Network (PPHSN), [3, 11] however gaps that exist globally [3, 16] are also prevalent and vary widely in the region [3]. Digitisation of health information systems including surveillance has been prioritised by health leaders in the region and is an opportunity to improve the availability and timeliness of surveillance data [12]. Laboratory based surveillance is limited by the availability of laboratory facilities and reagents that are influenced by global supply chains and the increased demands for testing during epidemics [4]. This affects the completeness of reporting in surveillance datasets and is a key limitation for producing accurate measures of disease incidence [3]. Furthermore, inconsistent recording of case location information [10, 41] is common, partly due to the fact that street addresses are not applicable in many PIC contexts. This limits the ability for spatial analyses of disease incidence pre- and post-intervention that contrasts populations in Wolbachia release areas from non-release areas. Community based surveillance has recently been introduced in Fiji for early warning of outbreaks, including dengue, however use of this data to assess disease incidence and risk distribution has not yet been explored [42].

Global limitations in entomological surveillance capacity [4, 16] also exist in PICs [11] and are a key challenge for collecting the data on mosquito populations needed for evaluation of vector control methods such as Wolbachia [10, 11]. Capacity building through the *Pacific Mosquito Surveillance Strengthening for Impact* (PacMOSSI) program in the Pacific provides a critical opportunity for PICs to develop entomological surveillance that can be integrated with disease surveillance routinely [11]. Incorporating capacity building for monitoring of novel interventions such as Wolbachia would be of benefit in countries where this has been introduced. Transitioning the focus of routine vector surveillance from immature mosquito indices (larval surveys) to adult mosquito monitoring, can enable regular monitoring of Wolbachia frequency to evaluate intervention impact [1]. Enhancing collaboration between health programs and partner organisations can improve support for local vector control and improve entomological surveillance if aligned with national priorities [1]. Citizen science initiatives for mosquito surveillance have been trialled in a range of settings [43] including the Solomon Islands [44, 45] with results suggesting this approach may be a feasible addition to arboviral disease vector surveillance providing appropriate resourcing, careful selection of participants and integration with existing surveillance are addressed.

#### Primary epidemiological research

Research studies on arboviral diseases in PICs exist mainly as outbreak reports and implementation studies of interventions. Randomized trials are considered the gold-standard for scientific inquiry in evaluating intervention efficacy however implementation of prospective studies in PICs may be costly, inefficient, and lack justification if efficacy has been demonstrated in other settings. More pragmatic approaches for providing the ‘best available’ scientific evidence in PICs can leverage existing data sources to provide sufficiently robust information more rapidly. For evaluation of the Wolbachia intervention in Pacific release sites, improving the quality of existing surveillance and clinical datasets can provide sufficient evidence for impact assessment using time series analyses or aggregate spatial comparisons of dengue incidence pre and post intervention.

#### Seroprevalence surveys

Seroprevalence surveys offer a valuable way to generate local evidence on pathogen transmission patterns, independent of surveillance data [46]. They have been used in several PICs to assess exposure to arboviral diseases, are especially useful where healthcare-seeking is limited, and have been recommended for countries considering vaccination [1, 47, 48]. Assessments of age-stratified dengue seroprevalence can support evaluations of Wolbachia’s impact on dengue transmission, and inform further implementation of Wolbachia and supplementary interventions including vaccination [1]. Improving the feasibility and cost-effectiveness of large-scale serosurveys in Pacific settings may be possible by leveraging use of dried blood spots and multiplex bead assays for integrated testing, though adequate financial, human, and laboratory resources are needed [47, 49].

#### Challenges in scientific evidence generation

The current paucity of published evidence on the public health impact of Wolbachia and other interventions in PICs [10, 14, 15] illustrate how the introduction of new well-intentioned public health interventions can inadvertently overwhelm existing capacities particularly where competing priorities for disease control abound. To offset systemic challenges, greater emphasis is needed from the outset on engaging decision makers and other stakeholders in identifying and generating the critical evidence for informing decision-making on the use of interventions, including their effectiveness, scalability and sustainability. More intervention is not necessarily ‘better’; instead, greater benefit may be achieved by allocating dedicated resources for more complete evaluation of activities in each PIC setting. Stakeholders and decision makers should be encouraged to promote systems for collating, archiving and disseminating the results of health programs and research that would best suit their own settings. Communication of results to communities involved is a critical priority not just for strengthening EIDM but for encouraging community involvement in decision making.

#### Tacit evidence

A considerable – and often overlooked – opportunity in strengthening the generation and use of evidence in Pacific health research is the large body of tacit evidence which exists in PICs. Eliciting community knowledge in PICs as a source of tacit evidence through methods such as *Talanoa* and *Tok Stori* present an opportunity to leverage culturally appropriate information for collective ownership of decisions and actions [50, 51]. *Talanoa* involves community-based conversations, open dialogue and deep listening to centre perceptions of health and acceptability of interventions around cultural ways of knowing [50, 52]. *Tok Stori* is a similar concept used in Melanesian contexts that has been used in social research [51]. Use of *Talanoa* has been shown to provide rich data for understanding Pacific community responses to COVID-19 interventions [50, 52]. Hence, tacit evidence for the Wolbachia intervention might best be elicited from communities in release areas, local experts and stakeholders using a *Talanoa* or *Tok Stori* methodology. This would provide culturally contextualized assessments of impact and acceptability to complement scientific data and inform future implementation strategies [50]. Incorporation of indigenous knowledge as part of the body of tacit evidence for arboviral disease control in PICs can strengthen public health approaches and direct prioritisation of primary research [25, 53]. Collaboration between stakeholders, health programs, working groups and communities is recommended to explore how tacit evidence for Wolbachia and other interventions might be generated and applied.

### Evidence application from knowledge to action

#### Knowledge translation pathways

Effective EIDM is dependent on knowledge translation pathways (KTPs) to enable the practical application of different types of evidence to influence policy and/or practice [18]. Many of the typical formal pathways and institutional structures for producing and using evidence in decision-making – such as guideline development, health technology assessment, health and economic modelling, and evaluation – do not exist or are still developing in PIC contexts [39, 40, 54]. However, the conceptual models for the translation of evidence to inform policy and practice outlined in the WHO EIDM guide provide guidance for considering how effective knowledge translation occurs that is broadly generalisable across different settings and local contexts. In most Pacific countries, pathways for evidence application include both ‘push efforts’ whereby the generators of research knowledge create key messages that are ’pushed’ to policy makers [17, 39] and ‘user-pull’ models whereby policy makers can access repositories of information. Scientific evidence for the Wolbachia intervention in Pacific sites generated by the WMP has been shared with local stakeholders using knowledge driven approaches rather than problem-solving approaches commissioned by decision makers. This is a common requirement for health programs and research initiatives that are supported by external funding. Strengthening communication between decision/policy makers, researchers and communities for evaluation activities may lead to more locally driven problem-solving approaches for knowledge translation. The recent formation of the Pacific Vector Network [11] is an encouraging development that can provide a regional KTP for arboviral disease interventions that supports knowledge translation at national and regional level.

A distinct advantage of Pacific health systems is their relative simplicity and size which can more easily accommodate continuous interactions and collaborations between stakeholders involved in EIDM compared to more complex systems [39–41]. There are often existing connections between evidence generators and users (people often know each other), which can be leveraged to help facilitate streamlined engagement and simpler pathways for uptake of evidence into decision making. Enhancing connections between the creators and users of evidence, including communities involved, is key to building better understanding of the ‘best possible’ decision for each context [17]. Specific policy dialogue opportunities for arboviral disease control have not yet emerged but may occur with strengthening of existing partnerships and increased recognition of the need for regular exchanges between programs, researchers, communities, and decision makers. Pacific cultures emphasise connections and relationality [52] hence encouraging cultural frameworks of connection to build opportunities for policy dialogues regarding disease control would strengthen initiatives in this area. The importance of integrating community values and perspectives in decision making and policy creation cannot be overstated and appropriate methodologies to elicit these should be driven by the communities involved.

#### Cross sectoral approaches

Enhanced EIDM in PICs can be achieved through cross-sectoral policies and actions for arboviral disease control that align with global strategies, [4] regional priorities for action [12, 16] and the ‘healthy islands’ vision [12, 55]. This vision supports holistic approaches to address broad health determinants such as recent One Health and planetary health programs in PICs [55]. The Revitalising Informal Settlements and their Environments (RISE) and Watershed Interventions for systems Health in the Pacific (WISH Pacific) collect comprehensive environmental, animal and health data and have developed cross-sectoral networks for intervention implementation and evaluation [41, 56]. Leveraging these initiatives for cross-sectoral collaboration can enhance EIDM efficiency and impact, particularly by facilitating integrated knowledge translation networks. For interventions like Wolbachia, this approach could expand the existing scope of evaluations to inform broader health goals.

## Conclusions

Strengthening the generation, application and utilisation of local evidence on the effectiveness of public health interventions for arboviral disease control is critical for PICs and will promote accountability to populations affected. The WHO framework for EIDM provides a useful guide to improve the generation and use of evidence in PICs with pragmatic consideration of the local contexts. Despite existing challenges in disease surveillance and a limited body of scientific evidence, Pacific countries can achieve effective decision making to progress health with relatively more efficiency than more complex settings. Eliciting tacit evidence through methods such as Talanoa and Tok Stori and leveraging the closer connections between evidence generators and users can optimise use of contextually appropriate evidence for decision making. Improvements in health information systems will improve the utility and timeliness of data for use in intervention evaluations. It is recommended that program implementation should prioritize inclusion of impact evaluation as an integral part of program design, with considerations of health system capacities, community acceptability, scalability and affordability at the design phase of intervention programs.

Mapping existing evidence using the evidence funnel for priority public health issues is useful for identifying research gaps. Pacific leadership in efforts to strengthen EIDM must be supported to promote sustainability and ensure that methodologies are contextually appropriate. In addition, providing evidence informed measures of impact to populations affected must be emphasised along with inclusion of communities in decision making. Centring community voices and experiences using culturally appropriate methods is critical in this decision-making process. Cross-sectoral approaches for health interventions can offset health system limitations and provide a broader range of evidence for integrated policy actions for health in Pacific countries. Opportunities for policy dialogues between policy makers, health programs, researchers and communities should be encouraged to identify opportunities for using evidence in different stages of the policy cycle and to determine the most useful evidence products. This could hasten progress for informed decision making even as sources of evidence are being compiled. This approach could provide a foundation for strengthening the creation and application of evidence in Pacific health programs and build collaborations to support future funding proposals. Pacific countries can benefit from utilising the WHO framework for EIDM in charting a course towards improved use of evidence for arboviral disease control and providing increased transparency in decision making for Pacific populations.

## Data Availability

No datasets were generated or analysed during the current study.

## Abbreviations

EIDM: Evidence informed decision-making
FNU: Fiji National University
KTP: Knowledge Translation Pathway
PacMOSSI: Pacific Mosquito Surveillance Strengthening for Impact
PICs: Pacific Island countries
WHO: World Health Organization
WISH: Watershed Interventions for Systems Health
WMP: World Mosquito Program
RISE: Revitalising Informal Settlements and their Environments
